# Social isolation and loneliness as risk factors for the progression of frailty: the English Longitudinal Study of Ageing

**DOI:** 10.1093/ageing/afx188

**Published:** 2017-12-22

**Authors:** Catharine R Gale, Leo Westbury, Cyrus Cooper

**Affiliations:** 1 MRC Lifecourse Epidemiology Unit, University of Southampton, Southampton, UK; 2 Centre for Cognitive Ageing & Cognitive Epidemiology, Department of Psychology, University of Edinburgh, Edinburgh, UK

**Keywords:** loneliness, social isolation, frailty, longitudinal, cohort, older people

## Abstract

**Background:**

loneliness and social isolation have been associated with mortality and with functional decline in older people. We investigated whether loneliness or social isolation are associated with progression of frailty.

**Methods:**

participants were 2,817 people aged ≥60 from the English Longitudinal Study of Ageing. Loneliness was assessed at Wave 2 using the Revised UCLA scale (short version). A social isolation score at Wave 2 was derived from data on living alone, frequency of contact with friends, family and children, and participation in social organisations. Frailty was assessed by the Fried phenotype of physical frailty at Waves 2 and 4, and by a frailty index at Waves 2–5.

**Results:**

high levels of loneliness were associated with an increased risk of becoming physically frail or pre-frail around 4 years later: relative risk ratios (95% CI), adjusted for age, sex, level of frailty and other potential confounding factors at baseline were 1.74 (1.29, 2.34) for pre-frailty, and 1.85 (1.14, 2.99) for frailty. High levels of loneliness were not associated with change in the frailty index—a broadly based measure of general condition—over a mean period of 6 years. In the sample as a whole, there was no association between social isolation and risk of becoming physically frail or pre-frail, but high social isolation was associated with increased risk of becoming physically frail in men. Social isolation was not associated with change in the frailty index.

**Conclusion:**

older people who experience high levels of loneliness are at increased risk of becoming physically frail.

## Introduction

Social relationships are important for health [1]. Most such research has focused on social isolation or loneliness. Social isolation is defined objectively using criteria such as having few contacts, little involvement in social activities and living alone. Loneliness is a subjective feeling of dissatisfaction with one’s social relationships. Both social isolation and loneliness have been linked with increased mortality [2–4], incident heart disease [5, 6] and functional decline [7, 8]. Social isolation and loneliness tend to be correlated, albeit weakly [9].

Frailty is a clinical syndrome whose main feature is heightened vulnerability to stressors due to lowered physiological reserves, decline in the ability to maintain homoeostasis and impairments in multiple systems [10]. There are two principal models. In Fried’s phenotype model, frailty is based on three or more components of poor grip strength, slow walking speed, low physical activity, exhaustion and unintentional weight loss [11]. The frailty index, or cumulative deficit model, defines frailty in terms of the accumulation of ‘deficits’ (symptoms, signs, diseases and disabilities). A frailty index score reflects the proportion of potential deficits present [12]. The phenotype model defines frailty in physical terms, whereas the cumulative deficit model uses a broader definition of frailty.

Frailty—defined either using the Fried phenotype or a frailty index—is associated with loneliness and social isolation [13–15]. The cause of these cross-sectional associations is uncertain. One possibility is that being frail leads to increases in loneliness and social isolation. There is some prospective evidence in support of this, but only as regards loneliness [15]. Another possibility is that greater loneliness and social isolation leads to increases in frailty. To our knowledge, no prospective study has investigated whether loneliness is a risk factor for change in frailty status, and in the only such study to examine social isolation’s relationship with future frailty, no association was found after adjustment for comorbidity [16]. Both loneliness and social isolation have been associated prospectively with decline in gait speed [7]. Gait speed is one of the five components of the phenotype of physical frailty [11], but there is insufficient evidence—or consensus—that it, or other single assessments, can reliably identify the syndrome of frailty [10, 17].

We used data from the English Longitudinal Study of Ageing (ELSA) to investigate whether social isolation and loneliness were independent risk factors for change in frailty status. Our secondary objective was to investigate whether greater frailty increased the likelihood of high levels of loneliness or social isolation in the future. We characterised frailty using both the Fried phenotype and a frailty index to assess whether relationships with social isolation or loneliness were consistent regardless of how frailty was defined.

## Methods

For a full description of the methodology, see Appendix 1. We provide a summary below.

### Participants

The sample for ELSA was based on participants aged ≥50 years in the Health Survey for England [18]. The initial survey took place in 2002–3. Subsequent data collection has taken place at 2-year intervals. The current study uses data from Waves 2–5.

### Measures

#### Loneliness

Participants completed the short version of the Revised UCLA loneliness scale [19], which enquires about frequency of feeling left out, isolated, and lacking companionship. Scores range from 3 to 9. Higher values indicate greater loneliness. Scores were positively skewed so for the regression analyses we categorised participants according to whether their score was low (3) average (4 or 5) or high (≥6).

#### Social isolation

We created a score for social isolation by giving one point for each of: being unmarried or not cohabiting, having less than monthly contact (whether face-to-face, written, or telephone) with each of children, other members of the family, and friends, and not being a member of organisations such as religious groups, evening classes, social groups or residents associations. Scores ranged from 0 to 5. Higher values indicate greater social isolation. Scores were positively skewed so for the regression analyses we categorised participants according to whether their score was low (0) average (1) or high (≥2).

#### Frailty

Frailty was assessed in two ways, as described previously [20]. Data from Waves 2–5 were used to derive a frailty index. A frailty index can be constructed from different numbers of variables [12]. It is recommended that at least 30 deficits are included [21]. Our index was made up of 52 deficits. The number of deficits present is added and then divided by the total number of deficits considered. Scores range from 0 to 1. Higher values indicate greater frailty. We used data from Waves 2 and 4 to derive the phenotype of physical frailty [11].

#### Covariates

Age, socioeconomic position, educational attainment, depressive symptoms and smoking at baseline were viewed as potential confounding variables. In models of the Fried phenotype of frailty, we also adjusted for number of chronic physical diseases at baseline.

### Analytical sample

In total, 6,183 core cohort members aged ≥60 participated in Wave 2. Of those, 3,505 were re-interviewed at Waves 3–5 and had sufficient data to allow the derivation of frailty index scores. Analyses of social isolation and loneliness in relation to change in the frailty index are based on 2,817 (80%) participants with complete data. Of the 6,183 core cohort members aged ≥60 who participated in Wave 2, 2,824 were re-assessed by a nurse at Wave 4 and had data on the frailty phenotype at both Waves. Analyses of social isolation and loneliness in relation to change in the frailty phenotype are based on 2,346 (83%) participants with complete data.

### Statistical analysis

Frailty index scores were positively skewed and were log-transformed for analysis, after the addition of 0.01 to avoid logarithms of zero. The frailty index change measure was characterised by fitting sex-specific linear mixed effects models with random intercepts and slopes for the frailty index score over the four time points. Sex-specific standard deviation scores for the random slopes were used as the measure of change in the frailty index score. This measure of change was weakly correlated with the baseline frailty index score among men (*r* = −0.26) and women (*r* = −0.20).

We used rank order correlations to examine loneliness and social isolation in relation to other characteristics. We used linear regression to calculate regression coefficients for change in frailty index score between Waves 2 and 5 according to baseline social isolation and loneliness. Estimates are adjusted for age and sex, and further adjusted for other covariates. We used multinomial logistic regression to derive relative risk ratios for becoming physically frail or pre-frail by Wave 4, according to baseline social isolation and loneliness. Estimates are shown adjusted for age, sex and the number of components of the phenotype that were present at baseline, and further adjusted for other covariates.

## Results

Table 1 shows the baseline characteristics of the sample and their rank order correlation with social isolation and loneliness. Being more socially isolated or lonelier was associated with being older, less educated, less wealthy, having more depressive symptoms, more chronic physical disease, being a smoker, having more components of the phenotype of frailty and a higher frailty index score. Being female was associated with greater loneliness and with slightly greater social isolation, though the latter relationship was of borderline significance (*P* = 0.066). There was a modest correlation between social isolation and loneliness (rho = 0.237) that did not differ between the sexes (*P* = 0.184). Participants who were physically frailer at baseline tended to have a higher frailty index score (rho = 0.40).

**Table 1.** Baseline characteristics of the participants and their rank order correlations with social isolation and loneliness scores (*n* = 2,817)^**a**^

Characteristic	Mean (SD), median (IQR) or No. (%)	Correlation with social isolation	Correlation with loneliness
Age (yrs), mean (SD)	69.3 (6.9)	0.105***	0.096***
Female, *n* (%)	1,604 (56.9)	0.035	0.110***
Household wealth (£), median (IQR)	207,300 (114,000–358,500)	−0.214***	−0.194***
Educational qualifications		−0.128***	−0.127***
No qualifications, *n* (%)	967 (34.3)		
Social isolation, median (IQR)	1 (0–2)	–	0.237***
Loneliness, median (IQR)	3 (3–5)	0.254***	–
Depressive symptoms, median (IQR)	0 (0–1)	0.069***	0.310***
Current smoker, *n* (%)	297 (10.5)	0.117***	0.070***
Number of chronic physical illnesses, median (IQR)	1 (0–2)	0.086***	0.161***
No. of components of frailty phenotype present at baseline, median (IQR)^a^	0 (0–1)	0.108***	0.231***
Frailty index at baseline, median (IQR)	0.146 (0.108–0.216)	0.120***	0.287***

^a^Descriptive data on the Fried phenotype of frailty are based on 2,346 participants. ***P < 0.001, **P < 0.01, *P < 0.05

The baseline prevalence of physical frailty or pre-frailty was 5.3% and 38.5%, respectively. By the Wave 4 follow-up, around 4 years later, prevalence had increased to 11.2% and 41%, respectively. Overall change in the frailty index from baseline to Wave 5, around 6 years later, was slight, with the median (IQR) score changing from 0.146 (0.108–0.216) at baseline to 0.142 (0.101–0.224), although there was considerable individual variation in change over time.

Table 2 shows relative risk ratios (RRR) (95% confidence intervals) for becoming physically frail or pre-frail at Wave 4, given level of frailty at baseline, according to social isolation and loneliness at baseline. In the model adjusted for age, sex and number of components of frailty present at baseline, participants who scored high for social isolation had an increased risk of becoming physically frail compared to those with low scores. High scores for social isolation were also associated with an increased risk of becoming pre-frail. These associations were attenuated and no longer significant after further adjustment for other covariates.

**Table 2:** Relative risk ratios (95% confidence intervals) of pre-frailty or frailty at Wave 4 according to social isolation or loneliness at baseline (*n* = 2,346)

Social isolation or Loneliness	RRR (95% CI), adjusted for age, sex & number of components of frailty present at baseline		RRR (95% CI), further adjusted for education, household wealth, depressive symptoms, chronic physical illness & smoking status at baseline	
	Pre-frail	Frail	Pre-frail	Frail
Social isolation				
Low (*n* = 782)	Reference	Reference	Reference	Reference
Average (*n* = 910)	1.12 (0.90, 1.38)	1.55 (1.04, 2.29)	0.92 (0.73, 1.15)	0.88 (0.57, 1.36)
High (*n* = 654)	1.47 (1.16, 1.86)**	2.00 (1.32, 3.04)**	1.19 (0.93, 1.53)	1.12 (0.70, 1.78)
Loneliness				
Low (*n* = 1,312)	Reference	Reference	Reference	Reference
Average (*n* = 647)	1.11 (0.90, 1.36)	1.42 (0.98, 2.06)	1.05 (0.84, 1.32)	1.19 (0.79, 1.78)
High (*n* = 387)	1.91 (1.45, 2.51)***	2.95 (1.95, 4.47)***	1.74 (1.29, 2.34)***	1.85 (1.14, 2.99)*

***P < 0.001, **P < 0.01, *P < 0.05. SD, standard deviation. Relative risk ratios obtained from multinomial logistic regression models.

All estimates are weighted to reduce potential bias due to attrition.

We found significant differences in risk of physical frailty and pre-frailty between those who gained high scores for loneliness and those who gained low scores. These increases in risk remained statistically significant, although attenuated, after full adjustment for potential confounding factors: compared to people who had a low score for loneliness, the multivariable-adjusted relative risk ratios (95% CI) for pre-frailty and frailty respectively among those with a high score for loneliness were 1.74 (1.29, 2.34) and 1.85 (1.14, 2.99) respectively.

As women tended to be lonelier and slightly more socially isolated than men, we investigated whether associations between levels of social isolation or loneliness and physical frailty differed between the sexes. The association between high social isolation and risk of becoming physically frail, though not pre-frail, differed between the sexes (*p* for interaction term = 0.01). No significant associations were found in women, but men with high scores for social isolation had an increased risk of becoming physically frail that was of borderline significance: the RRR (95% CI) adjusted for all covariates was 2.01 (1.00, 4.05). The associations between loneliness and risk of becoming physically frail or pre-frail did not differ between the sexes (*p* for interaction terms all >0.13).

Table 3 shows regression coefficients for change in the frailty index from baseline to Wave 5, around 6 years later, according to social isolation and loneliness at baseline. There were no significant associations between either social isolation or loneliness and change in frailty index. These associations did not differ by sex (*p* for interaction terms all >0.3).

**Table 3.** Regression coefficients (95% confidence intervals) for change in frailty index (per SD) by Wave 5 according to social isolation or loneliness at baseline (*n* = 2,817)

Social isolation or Loneliness	Regression coefficient (95% CI), adjusted for age and sex	Regression coefficient (95% CI), further adjusted for education, household wealth, depressive symptoms, & smoking status at baseline
Social isolation		
Low (*n* = 931)	Reference	Reference
Average (*n* = 1,087)	0.038 (−0.046, 0.122)	0.051 (−0.034, 0.136)
High (*n* = 799)	−0.023 (−0.114, 0.068)	−0.008 (−0.100, 0.086)
Loneliness		
Low (*n* = 1,545)	Reference	Reference
Average (*n* = 769)	−0.008 (−0.091, 0.074)	0.020 (−0.065, 0.104)
High (*n* = 503)	−0.074 (−0.171, 0.024)	−0.007 (−0.111, 0.096)

The frailty change measure was obtained by fitting sex-specific linear mixed effects models for the frailty index score over Waves 2, 3, 4 and 5. Standard deviation scores for the random slopes were used as the measure of change. A positive regression coefficient indicates increase or reduced loss in frailty index and a negative coefficient reflects accelerated loss.

All estimates are weighted to reduce potential bias due to attrition.

We examined whether greater frailty at baseline increased the likelihood of experiencing high levels of loneliness or social isolation in the future. Greater physical frailty and having a higher frailty index at baseline were each associated with an increased risk of reporting high levels of loneliness in subsequent follow-ups, after adjustment for age, sex, baseline level of loneliness and other covariates (see Appendix 2). Findings linking either measure of frailty with increases in social isolation were slightly less consistent: after adjustment for age, sex, baseline social isolation and other covariates, baseline physical frailty was associated with an increased risk of high levels of social isolation two years later at Wave 3, but not at subsequent follow-ups, and baseline frailty index was associated with increased risk of social isolation at Waves 4–5, but not at Wave 3 (see Appendix 3).

## Discussion

To our knowledge, there have been no prospective studies of loneliness as a risk factor for frailty. However, prospective findings linking high levels of loneliness with decline in gait speed [7] or mobility [8], and increased difficulties with activities of daily life [7], or upper extremity tasks [8] suggest that loneliness may increase the likelihood of sarcopenia, an age-related syndrome characterised by loss of skeletal muscle mass and strength [22]. Sarcopenia is a major contributor to risk of functional decline and physical frailty [23]. The aetiology of sarcopenia is multifactorial, involving comorbidity, inflammation, insulin resistance, changes in endocrine function, nutritional deficiencies and low physical activity [24]. The latter may be one mechanism underlying the association between loneliness and progression of physical frailty. Lonely people are more likely to be inactive [25, 26], and such inactivity increases the risk of physical frailty [27, 28]. Another potential mechanism may be diet. More socially engaged older people tend to have a higher quality diet [29].

Studies of frailty risk factors usually use one model of frailty only, most commonly a frailty index or the phenotype of physical frailty [10]. Here, we used both models and found that although loneliness was a significant predictor of progression of physical frailty, it was not a risk factor for change in the frailty index. We found a similar pattern in an earlier study of this cohort in that having a more positive attitude to ageing was associated with lower risk of becoming physically frail, but was not associated with change in a frailty index [20]. One explanation may lie in the broader definition of frailty inherent in the cumulative deficit model which describes the general state or condition of an individual rather than a specific medical syndrome [30]. Our findings highlight the fact that the two principal models of frailty do not have the same risk factors.

Evidence on whether social isolation increases risk of frailty is sparse. One study found that persistent social isolation over two to three waves of follow-up was associated with an increased risk of being frail (defined as problems in two or more domains: physical, cognitive, sensory or nutritive) but numbers of cases were small and the association became non-significant after adjustment for physical illness [16]. Here, in the sample as a whole, high social isolation was not associated with increased risk of becoming physically frail or pre-frail once we controlled for confounding factors. Social isolation was not linked with progression of frailty as measured by a frailty index. Our findings on social isolation and physical frailty contrast with those of Shankar *et al.* who found that high social isolation was a risk factor for decline in gait speed [7]. This inconsistency is not unexpected given that change in physical frailty status is likely to involve varying degrees of change in all five components of the phenotype, not just gait speed.

We found evidence that the relationship between loneliness and frailty is bidirectional. Greater frailty increased the likelihood of high levels of loneliness in the future. This is consistent with observations in the Longitudinal Ageing Study Amsterdam [15]. In the Amsterdam study, frailty was not associated with decline in social network size or in emotional or instrumental support [15]. Here, we found indications that greater frailty—as measured by the frailty index in particular—was associated with increased risk of experiencing more social isolation subsequently. Our findings suggest that frailty may lead to increased social isolation, but not *vice versa.*

Strengths of our study include the large sample size and the fact that ELSA is designed to be representative of the community-dwelling English population aged ≥50. The study has limitations. Those excluded tended to be frailer, lonelier and more isolated than those who were included. Our findings may underestimate the true associations between loneliness or social isolation and change in frailty status. We may not have controlled adequately for depressive symptoms. These were assessed with the short version of the CES-D, three items of which we excluded due to potential overlap with the loneliness measure or because they were used as indicators of the exhaustion component of the Fried phenotype.

In this prospective study, high levels of loneliness were associated with increased risk of physical frailty. By contrast, loneliness was not linked with the rate at which a frailty index—a broadly based measure of general condition—changed over time.

**Key Points**

- Loneliness and social isolation have been linked with premature mortality and with functional decline in older people.
- We found that high levels of loneliness, but not of social isolation, increased the risk of becoming physically frail.
- Neither loneliness nor social isolation were associated with the rate of change in a more broadly defined frailty index.

## Supplementary Material

Supplementary Data

Supplementary Data

Supplementary Data
