# Supplementary material for: Social isolation and loneliness as risk factors for the progression of frailty: the English Longitudinal Study of Ageing
Source: Age Ageing. 2017 Dec 22;47(3):392–7. doi: 10.1093/ageing/afx188 (PMC5920346; doi:10.1093/ageing/afx188)
Supplement: Supplementary Data [file aa-17-0294-file002.docx]

**Appendix 1**

**METHODS**

*Participants*

The sample for ELSA was based on participants aged ≥50 years in the Health Survey for England.[1] The initial survey took place in 2002-3. Subsequent data collection has taken place at two-year intervals. Refreshment samples were added at Waves 3-4 to maintain the representation of people aged 50-75. The current study uses data from Waves 2-5. Ethical approval was obtained from the London Multicentre Research Ethics Committee. Participants gave written informed consent.

*Measures*

Loneliness

Participants completed the three-item short version of the Revised UCLA loneliness scale,[2] which enquires about frequency of feeling left out, isolated from others, and lacking companionship. There were three response options: hardly ever or never, some of the time, often. Cronbach alpha for the items in these data was 0.83, indicating good internal consistency. Ratings were summed to produce a score ranging from 3 to 9. Higher values indicate greater loneliness. Scores were positively skewed so for the regression analyses we categorized participants according to whether their score was low (3) average (4 or 5) or high (≥6), as well as using the score as a continuous variable.

Social isolation

Participants responded to items on social contact. As in previous studies,[3] we created a score for social isolation by giving one point for each of the following: being unmarried or not cohabiting, having less than monthly contact (whether face-to-face, written, or telephone) with each of children, other members of the family, and friends, and not being a member of organizations such as religious groups, evening classes, social groups, or residents associations. Scores ranged from 0 to 5. Higher values indicate greater social isolation. Scores were positively skewed so for the regression analyses we categorized participants according to whether their score was low (0) average (1) or high (≥2), as well as using the score as a continuous variable.

Frailty

We characterised frailty with a frailty index and with the Fried phenotype, as described previously.[4] Data from Waves 2-5 were used to derive a frailty index. A frailty index can be constructed using different numbers or types of variables.[5] It is recommended that at least 30 deficits are included to ensure precision.[6] The variables should be associated with health status, represent conditions that become more common with age, and cover different systems.[6] Our frailty index was made up of 52 deficits (see Table), assessed identically at each wave (tests used to assess cognitive function changed at wave 6 so we used data from waves 2-5 only). The number of deficits present is summed and then divided by the total number of deficits considered, which gives a frailty index score ranging from 0 to 1. Higher values indicate greater frailty.

**Table: Deficits included in the frailty index and how they were scored**

| **Type of deficit** | **Deficit** |
| --- | --- |
| Problems with… | Dressing, including putting on shoes or socks (Yes=1, No=0) |
|  | Bathing or showering (Yes=1, No=0) |
|  | Getting in or out of bed (Yes=1, No=0) |
|  | Eating, such as cutting up food (Yes=1, No=0) |
|  | Using the toilet, including getting up or down (Yes=1, No=0) |
|  | Using a map to figure out how to get around in a strange place (Yes=1, No=0) |
|  | Preparing a hot meal |
|  | Shopping for groceries |
|  | Making telephone calls (Yes=1, No=0) |
|  | Taking medications (Yes=1, No=0) |
|  | Doing work round the house or garden (Yes=1, No=0) |
|  | Managing money (Yes=1, No=0) |
|  | Walking across the room (Yes=1, No=0) |
|  | Walking a 100 yards (Yes=1, No=0) |
|  | Sitting for about 2 hours (Yes=1, No=0) |
|  | Getting up from a chair after sitting for long periods (Yes=1, No=0) |
|  | Climbing a single flight of stairs (Yes=1, No=0) |
|  | Stooping, kneeling or crouching (Yes=1, No=0) |
|  | Reaching or extending your hands above shoulder level (Yes=1, No=0) |
|  | Pulling or pushing large objects like a living room chair (Yes=1, No=0) |
|  | Lifting or carrying weights over 10 pounds like a heavy bag of groceries (Yes=1, No=0) |
|  | Picking up a 5p coin from a table (Yes=1, No=0) |
| Doctor has diagnosed: | Angina (Yes=1, No=0) |
|  | A heart attack (including myocardial infarction or coronary thrombosis) (Yes=1, No=0) |
|  | Congestive heart failure (Yes=1, No=0) |
|  | A heart murmur (Yes=1, No=0) |
|  | An abnormal heart rhythm (Yes=1, No=0) |
|  | Diabetes or high blood sugar (Yes=1, No=0) |
|  | A stroke (cerebrovascular disease) (Yes=1, No=0) |
|  | Chronic lung disease such as chronic bronchitis or emphysema (Yes=1, No=0) |
|  | Arthritis (including osteoarthritis or rheumatism) (Yes=1, No=0) |
|  | Osteoporosis, sometimes called thin or brittle bones (Yes=1, No=0) |
|  | Cancer or a malignant tumour (excluding minor skin cancers) (Yes=1, No=0) |
|  | Parkinson’s disease (Yes=1, No=0) |
|  | Any emotional, nervous or psychiatric problems (Yes=1, No=0) |
|  | Alzheimer’s disease (Yes=1, No=0) |
|  | Dementia, organic brain syndrome, senility or other serious memory problem (Yes=1, No=0) |
| Eyesight | (Excellent = 0, Very Good = 0.25, Good = 0.5, Fair = 0.75, Poor or blind = 1) |
| Hearing | (Excellent = 0, Very Good = 0.25, Good = 0.5, Fair = 0.75, Poor = 1) |
| Falls | Fallen in the last year (Yes=1, No=0) |
| Hip fracture | Yes=1, No=0 |
| Joint replaced | Yes=1, No=0 |
| Correct day of month given | Yes=0, No=1 |
| Correct year given | Yes=0, No=1 |
| Correct month given | Yes=0, No=1 |
| Cognitive function | Total score on composite measure* divided into quartiles (1 (lowest scores)=1, 2=0.6, 3=0.3, 4 (highest scores=0) |
| Often troubled by severe pain | Often troubled by severe pain (Yes=1, No=0) |
| Pain while walking | Yes=1, No=0 |
| Incontinence | Lost any urine beyond your control in last 12 months (Yes=1, No=0) |
| Self-rated health | (Excellent = 0, Very Good = 0.25, Good = 0.5, Fair = 0.75, Poor = 1) |

*The cognitive tests used were prospective memory, verbal fluency, verbal memory (immediate and delayed), and letter cancellation task. A standardized overall measure of general cognitive ability was generated by applying principal components analysis to the test scores and extracting (and calculating a score for each person on) the first unrotated principal component that reflects the variance shared among the tests taken.

The Fried phenotype of physical frailty was constructed using data from Waves 2 and 4.[7] Height and weight were measured with a portable stadiometer and electronic scales respectively. Body mass index (BMI) was calculated as weight (in kilograms)/height (in metres)^2^ . Handgrip strength was measured three times on each side using a dynamometer; the maximum of these measurements was used for analysis. Gait speed was assessed in participants aged 60 and over by measuring the time taken to walk a distance of 8 feet at usual pace; the timed walk was repeated and the mean of the two measurements was calculated. Participants responded to three questions about the frequency with which they did vigorous, moderate or mild exercise. We ranked the combinations of responses to these questions according to the amount and intensity of exercise involved to provide an estimate of usual physical activity. Participants completed the eight-item Center for Epidemiologic Studies Depression Scale (CES-D).[1] We operationalized the Fried phenotype criteria using definitions very similar to those used in the original phenotype of frailty studies [2,3]: weight loss was defined as *either* loss of ≥10% of body weight since the initial survey (for frailty at Wave 2) or since Wave 2 (for frailty at Wave 4), *or* current BMI <18.5 kg/m^2^; weakness was defined as maximum grip strength in the lowest 20% of the distribution, after taking sex and BMI into account; exhaustion was considered present if the participant gave a positive response to either of the CES-D items ‘Felt that everything I did was an effort in the last week’ or ‘Could not get going in the last week’; slow walking speed was defined as a walking speed in the lowest 20% of the distribution, after taking account of sex and height; and low physical activity was defined as physical activity in the lowest sex-specific 20% of the distribution. Frailty was defined by the presence of three or more criteria. Pre-frailty was defined by the presence of one or two criteria.

Covariates

We chose age, socioeconomic position (measured by household wealth), educational attainment, depressive symptoms and smoking at Wave 2 (baseline) as potential confounding variables. In models where change in the Fried phenotype of frailty was the outcome, we also adjusted for number of chronic physical diseases and number of components of the phenotype present at baseline. (Diagnoses of disease were among the ‘deficits’ used to derive the frailty index so were not included as covariates in models where the frailty index was the outcome.)

Socioeconomic position was indexed by total household wealth, including savings and investments, value of any property or business assets, net of debt, excluding pension assets. Household wealth has been identified as the most accurate indicator of long-term socioeconomic circumstances in ELSA.[8] Participants were asked about their educational qualifications. Highest educational qualification obtained was classified into seven categories; we reverse-coded these so that higher categories indicted greater educational attainment (7= National Vocational Qualification level 4 (NVQ4)/NVQ5/Degree or equivalent, 6= Higher education below degree, 5= NVQ3/GCE A Level equivalent, 4= NVQ2/GCE O Level equivalent, 3= NVQ1/CSE other grade equivalent, 2= Foreign/other, 1=No qualification. Symptoms of depression were assessed using the eight-item version of the Center for Epidemiologic Studies Depression Scale (CES-D).[9] As two items from the scale were used as an indicator of exhaustion when deriving the physical frailty phenotype and one item asked about loneliness, we calculated a total CES-D score after excluding these items.

*Analytical sample*

In total, 6183 core cohort members aged ≥60 participated in Wave 2. Of those, 3505 were re-interviewed at Waves 3, 4, and 5 and had sufficient data (on at least 30 out of a potential 52 variables) to allow the derivation of a frailty index at each wave. The analyses of social isolation and loneliness in relation to change in the frailty index are based on 2817 (80%) participants who had complete data on all variables. Of the 6183 core cohort members aged ≥60 who participated in Wave 2, 2,824 were re-assessed by a nurse at Wave 4 and had data on the frailty phenotype at both Waves. The analyses of social isolation and loneliness in relation to change in the phenotype of frailty are based on 2346 (83%) participants who had complete data on all variables. Compared to participants who were in our main analytical sample (n=2817), those who were excluded due to loss to follow-up or missing data were older, frailer, less wealthy, less educated, lonelier, more socially isolated and had more chronic physical illnesses.

*Statistical analysis*

Frailty index scores were positively skewed and were log-transformed for analysis, after the addition of 0.01 to avoid logarithms of zero. The frailty index change measure was characterised by fitting sex-specific linear mixed effects models with random intercepts and slopes for the frailty index score over the four time points. Sex-specific standard deviation scores for the random slopes were used as the measure of change in the frailty index score. This measure of change was weakly correlated with the baseline frailty index score among men (r = -0.26) and women (r = -0.20).

We used rank order correlations to examine loneliness and social isolation in relation to other characteristics. We used linear regression to calculate regression coefficients for change in frailty index score between Waves 2 and 5 according to social isolation and loneliness at baseline’. Estimates are adjusted for age and sex, and then further adjusted for other covariates. We used multinomial logistic regression to derive relative risk ratios for becoming physically frail or pre-frail by Wave 4, given frailty status at Wave 2, according to social isolation and loneliness at baseline. Estimates are shown adjusted for age, sex and the number of components of the phenotype that were present at baseline, and then further adjusted for other covariates. All estimates were weighted using longitudinal weights supplied with the data to minimize bias due to attrition since Wave 1.

**References**

1. Steptoe A, Breeze E, Banks J, Nazroo J. Cohort Profile: The English Longitudinal Study of Ageing. Int J Epidemiol. 2013;42:1640-8.

2. Hughes ME, Waite LJ, Hawkley LC, Cacioppo JT. A short scale for measuring loneliness in large surveys - Results from two population-based studies. Research on Aging. 2004;26:655-72.

3. Steptoe A, Shankar A, Demakakos P, Wardle J. Social isolation, loneliness, and all-cause mortality in older men and women. Proc Natl Acad Sci U S A. 2013;110:5797-801.

4. Gale CR, Cooper C. Attitudes to Ageing and Change in Frailty Status: The English Longitudinal Study of Ageing. Gerontology. 2017.

5. Rockwood K, Mitnitski A. Frailty in relation to the accumulation of deficits. J Gerontol A Biol Sci Med Sci. 2007;62:722-7.

6. Searle SD, Mitnitski A, Gahbauer EA, Gill TM, Rockwood K. A standard procedure for creating a frailty index. BMC geriatrics. 2008;8:24.

7. Fried LP, Tangen CM, Walston J, Newman AB, Hirsch C, Gottdiener J, et al. Frailty in older adults: evidence for a phenotype. J Gerontol A Biol Sci Med Sci. 2001;56:M146-M56.

8. Banks J, Karlsen S, Oldfield Z. Socio-economic position. In: Marmot M, Banks J, Blundell R, Lessof C, Nazroo J, editors. Health, wealth and lifestyles of the older population in England. London: Institute of Fiscal Studies; 2003. p. 71-125.

9. Steffick DE, The HRS working group. Documentation of affective functioning measures in the Health and Retirement Study. HRS/AHEAD Documentation Report DR-005 [online report]. 2000 [updated 2000]; Available from: <http://hrsonline.isr.umich.edu/sitedocs/userg/dr-005.pdf>.
