# Supplementary material for: Social isolation and loneliness as risk factors for the progression of frailty: the English Longitudinal Study of Ageing
Source: Age Ageing. 2017 Dec 22;47(3):392–7. doi: 10.1093/ageing/afx188 (PMC5920346; doi:10.1093/ageing/afx188)
Supplement: Supplementary Data [file aa-17-0294-file003.docx]

**Appendix 2: Odds ratios (95% confidence intervals) for gaining high scores for loneliness at Waves 3, 4 or 5 according to physical frailty or frailty index score at baseline (Wave 2)**

|  | **High scores for loneliness at Wave 3** | | **High scores for loneliness at Wave 4** | | **High scores for loneliness at Wave 5** | |
| --- | --- | --- | --- | --- | --- | --- |
| **Frailty measures at baseline^1^** | **OR (95% CI), adjusted for age, sex & loneliness score at baseline** | **OR (95% CI), further adjusted for education, household wealth, & chronic physical illness^1^** | **OR (95% CI), adjusted for age, sex & loneliness score at baseline** | **OR (95% CI), further adjusted for education, household wealth, & chronic physical illness^1^** | **OR (95% CI), adjusted for age, sex & loneliness score at baseline** | **OR (95% CI), further adjusted for education, household wealth, & chronic physical illness^2^** |
| Fried phenotype |  |  |  |  |  |  |
| Not frail | Reference | Reference | Reference | Reference | Reference |  |
| Pre-frail | 1.20 (0.96, 1.51) | 1.13 (0.90, 1.43) | 1.51 (1.20, 1.91)*** | 1.45 (1.14, 1.84)** | 1.42 (1.13, 1.79)** | 1.37 (1.09, 1.74)** |
| Frail | 1.67 (1.17, 2.39)** | 1.48 (1.02, 2.16)* | 2.08 (1.40, 3.10)*** | 1.91 (1.26, 2.91)** | 1.63 (1.09, 2.45)* | 1.46 (0.96, 2.23) |
| Frailty index, per SD | 1.24 (1.13, 1.35) *** | 1.19 (1.08, 1.30) *** | 1.23 (1.13, 1.35) *** | 1.20 (1.10, 1.32) *** | 1.25 (1.16, 1.37) *** | 1.22 (1.12, 1.34) *** |

*******p<0.001, **p<0.01, *p<0.05. SD=standard deviation. Odds ratios obtained from logistic regression models.

^1^  Analyses of the Fried phenotype are based on the following numbers: n=3293 at wave 3, 2798 at wave 4 and n=2626 at wave 5. Analyses of the frailty index are based on the following numbers: n=4440 at wave 3, n=4333 at wave 4 and n= 4351 at wave 5.

^2^ Number of chronic physical illness was not used as a covariate when examining frailty index as a predictor of future loneliness because diagnoses of illness are part of the frailty index measure
