# Supplementary material for: Social isolation and loneliness as risk factors for the progression of frailty: the English Longitudinal Study of Ageing
Source: Age Ageing. 2017 Dec 22;47(3):392–7. doi: 10.1093/ageing/afx188 (PMC5920346; doi:10.1093/ageing/afx188)
Supplement: Supplementary Data [file aa-17-0294-file004.docx]

**Appendix 3: Odds ratios (95% confidence intervals) for gaining high scores for social isolation at Waves 3, 4 or 5 according to physical frailty or frailty index score at baseline (Wave 2)**

|  | **High scores for social isolation at Wave 3** | | | **High scores for social isolation at Wave 4** | | **High scores for social isolation at Wave 5** | |
| --- | --- | --- | --- | --- | --- | --- | --- |
| **Frailty measures at baseline^1^** | **OR (95% CI), adjusted for age, sex & social isolation score at baseline** | **OR (95% CI), further adjusted for education, household wealth, & chronic physical illness^1^** | | **OR (95% CI), adjusted for age, sex & social isolation score at baseline** | **OR (95% CI), further adjusted for education, household wealth, & chronic physical illness^1^** | **OR (95% CI), adjusted for age, sex & social isolation score at baseline** | **OR (95% CI), further adjusted for education, household wealth, & chronic physical illness^2^** |
| Fried phenotype |  |  |  | |  |  |  |
| Not frail | Reference | Reference | | Reference | Reference | Reference |  |
| Pre-frail | 1.44 (1.16, 1.77)*** | 1.38 (1.11, 1.71)** | | 1.45 (1.15, 1.81)** | 1.30 (1.03, 1.64)* | 1.37 (1.09, 1.71)** | 1.26 (1.00, 1.59) |
| Frail | 1.79 (1.23, 2.62)** | 1.67 (1.13, 2.47)* | | 1.85 (1.19, 2.88)** | 1.43 (0.91, 2.27) | 1.36 (0.87, 2.11) | 1.10 (0.69, 1.75) |
| Frailty index, per SD | 1.15 (1.05, 1.26)** | 1.09 (1.00, 1.20) | | 1.20 (1.09, 1.31)*** | 1.12 (1.02, 1.23)* | 1.22 (1.12, 1.34)*** | 1.16 (1.06, 1.27)** |

*******p<0.001, **p<0.01, *p<0.05. SD=standard deviation. Odds ratios obtained from logistic regression models.

^1^ Analyses of the Fried phenotype are based on the following numbers: n=2864 at wave 3, 2461 at wave 4 and n=2286 at wave 5. Analyses of the frailty index are based on the following numbers: n=3939 at wave 3, n=3863 at wave 4 and n= 38451 at wave 5.

^2^ Number of chronic physical illness was not used as a covariate when examining frailty index as a predictor of future loneliness because diagnoses of illness are part of the frailty index measure
